# 

**Published:** 1892-05-28

**Authors:** 


**Notices of Births, Deaths, and Marriages,** are inserted in this column at a charge of 2s. 6d. for an announcement not exceeding four lines.

### NOTICE TO CORRESPONDENTS.

All MS., Letters, Books for Review, and other matters intended for the Editor should be addressed The Editor, The Lodge, Porchester Square, London, W.

The Editor cannot undertake to return rejected M.S., even when accompanied by stamped directed envelope.

**Notice to Advertisers and Subscribers.**

All Advertisements, Orders for Copies of The Hospital, and Business Communications should be addressed to The Publisher, *not to the Editor*, The Hospital, 140, Strand, W.C.

The Cost of Subscription, post free, is as follows:—Per week, 2½d.; per quarter, 2s. 6d.; per half-year, 5s.; per annum, 8s. 6d. Foreign:—Per annum, 10s. 8d.; payable, in all cases, in advance.

**"Burdett's Hospital Annual," 1891—1892.**

Third year of publication. 600 pp. Boards, gilt lettering. A most complete guide to British and Colonial Hospitals, Dispensaries, Nursing and Convalescent Institutions, and Asylums. Price 3s. 6d. Published by The Scientific Press (Limited), 140, Strand, W.C.

**NOTICE.—The Index for Vol. XI. (ending March, 1892), is on sale at the Office of this Journal, price 2½d., post free.**

### Scraps and Gleanings.

Subscriptions amounting to £3,200 were received at the annual dinner of King's College Hospital.

The receipts of the West Ham Hospital for 1891 amounted to £3,033 15s. 8d., and the expenditure to £2,918 12s. 11d.

July 20th is the day fixed for the laying of the foundation stone of the new home in connection with the British Home for Incurables.

Influenza is reported to be raging in Trinidad and that there were recently 600 cases in Port of Spain alone, 10 per cent. of which proved fatal.

At the Hospital for Women, Bath, 87 indoor patients were admitted during the year. The attendance in the outdoor department was very large.

The sum of £28 14s. 3d. was realised by "The Mock Trial," given by the members of the Hull Law Students' Society, in aid of the Hull Royal Infirmary.

The Duchess of Westminster will distribute the Health Society's medals and certificates in the Reuben's Gallery, Grosvenor House, on Tuesday, at half-past three.

The Duke of Fife, President of the Hospital for Sick Children, Great Ormond Street, will take the chair at the annual court to be held at the hospital on the 31st inst.

A "Times" telegram reports that cholera in a severe epidemic form has appeared in the Cashmere Valley; 296 cases, with 146 deaths, were reported from Srinagar on Sunday.

There seems a great probability that the Birmingham Hospital Saturday Fund this year will amount to £12,000. Up to the 21st inst. £11,115 12s. 7d. had been paid into the bank.

Mr. Charles Welling, late of New York, has bequeathed £200 to the Bootle Hospital as a thankoffering for the kind attention and great benefits he experienced while lying ill in that institution.

Of the 2,382 patients received into the Devonshire Hospital, Buxton, during the past twelve months ending April 3rd, 1892, 2,231 were sent away benefited, although many of the cases were of a serious character.

The Iver Langley and Denham Cottage Hospital received 71 patients during the year ending March 31st. This is a larger number of patients than has been recorded in any previous year, and some of the cases were very serious.

The Duchess of Westminster recently opened an extension of the Belgrave Hospital for Children, Gloucester-street, S.W. In the past two years £700 has been collected for the institution by the working classes of the neighbourhood.

The Duke of Cambridge, owing to a bronchial attack, was unable to preside at the festival dinner in aid of the Clarence Memorial Fund of St. Mary's Hospital, Paddington. Subscriptions amounting to over £10,000, towards the £100,000 which is required, were announced.

During the past year, 536 patients were treated in the Mercy Hospital, Cork. The new departure, viz., that of ophthalmic surgery, showed a good promise for the future; 91 serious operations were performed, and 3,763 patients were examined and treated in the extern department.

It is stated that a legacy of £6,738 from Mr. James Nasmyth, and another of £1,000 from Miss L. A. Warrington, has been received by the Hospital for Sick Women, Soho Square, during the year, while the grants from the Hospital Saturday and Sunday Funds have greatly increased.

At the annual meeting of the Liddell Provident Dispensary, Yarrow, the Chairman stated that through lack of funds, it was the intention of the Committee to close the institution. During the eight years the dispensary has been carried on, it has not met with the support necessary to carry on the work.

Subscriptions and donations to the amount of £2,600 were announced during the evening of the thirty-sixth anniversary festival of the Royal Hospital for Incurables. According to the report, there are now upwards of 810 persons on the roll of the institution, the annual expenses of which are close upon £30,000.

The Mayoress of Birmingham on Saturday opened at Llandudno the Convalescent Home which has been established at that watering-place by the Birmingham Hospital Committee. The approximate cost is £10,000, £7,000 of which was subscribed by the Misses Stokes, after whom the institution has been named.

A general meeting of the Governors of the Hastings, St. Leonards, and East Sussex Hospital is to be held on the 30th inst., to consider a requisition for rescinding the 53rd rule, so as to throw open the office of physician and assistant physician to others than fellows or members of the Royal College of Physicians of London.

The annual report of the Society for Prevention of Cruelty to Children shows that the past year has been a period of the most remarkable general advance the society has ever known, the number of cases investigated being 8,324. The total expenditure was £26,000, which, with a deficit of £1,100 at the end of last year, accounted for an income of £27,000.

The following donations towards re-building of the Royal Ophthalmic Hospital, Moorfields, have already been promised: £1,000 from the surgical staff, £500 from the Goldsmiths' Company, 200 guineas from the Corporation of London, 210 guineas from Mr. John Deacon, 100 guineas from the Merchant Taylors' Company, and £100 from Sir John Lubbock, the President.

Through the operation of the Children's Country Holidays Fund over 25,000 children enjoyed the benefit of a fortnight's holiday in country cottages last year. The parents of the children enjoying the holidays provided by this fund contributed more than one-third of the charge per head, which, for a fortnight's holiday, including all expenses of transit and organisation, was only just over twelve shillings and sixpence.

### THE STUDENT OF MEDICINE.

[All communications for this Supplement should be addressed to The Editor of The Hospital, at 140, Strand, with the words, "Students' Column," written in the left hand top corner of the envelope.]

### APPOINTMENTS.

Anderson, W., M.B., C.M.Aberd., appointed Resident Assistant Medical Officer to the Aberdeen Royal Infirmary. Andrew, H., M.R.C.S.Eng., L.R.C.P.Lond., appointed House-Surgeon to the Devon and Exeter Hospital, Exeter. Atkins, J. F., L.R.C.P.Lond., M.R.C.S.Eng., appointed Assistant Medical Officer to the Birmingham Workhouse Infirmary. Barber, F. S., M.R.C.S.Eng., L.R.C.P.Lond., appointed Assistant Surgeon to the Bradford Infirmary. Berry, A. E., M.B., L.R.C.P.Lond., M.R.C.S., appointed Assistant Medical Officer of the Crumpsall Workhouse, Manchester. Bristowe, J. S., M.D., F.R.C.P.Lond., F.R.S., appointed Consulting Physician to the West End Hospital for Diseases of the Nervous System, Paralysis, and Epilepsy. Fitzgerald, G. C., B.A., M.B., B.C.Camb., appointed Medical Superintendent of the Kent County Asylum, Chartham. Heath, C., F.R.C.S.Eng., appointed Consulting Surgeon to the West End Hospital for Diseases of the Nervous System, Paralysis, and Epilepsy. Little, J. F., M.B. Cantab., M.R.C.P.Lond., appointed Lecturer and Demonstrator on Electro-Therapeutics and Massage at the West End Hospital for Diseases of the Nervous System, Paralysis, and Epilepsy. Macintosh, D., M.B., C.M.Aberd., appointed Resident Assistant Medical Officer to the Aberdeen Royal Infirmary. Matthews, A. A., L.D.S.R.C.S., appointed Honorary Dental Surgeon to the Bradford Infirmary. Pirie, W. R., M.B., C.M.Aberd., appointed Resident Assistant Medical Officer to the Aberdeen Royal Infirmary. Rust. J., M.A., M.B., C.M.Aberd., appointed Resident Assistant Medical Officer to the Aberdeen Royal Infirmary. Smith, H., M.B., B.S., appointed Medical Officer for the Workhouse of the Durham Union. Wightman, J. P., L.R.C.P.Lond., M.R.C.S., appointed Junior House-Surgeon to the Bradford Infirmary. Ann Augusta Wilson, M.D.Brux., L.R.C.P. and S.Edin., has been appointed Resident Medical Officer, to the Clapham Maternity Hospital, London. Ellen Berthon, M.B.Lond., has been appointed Physician to the Out-Patient Department, Wandsworth Road, of Clapham Maternity Hospital, London.

### VACANCIES.

### Resident Medical Officers.

The following vacancies are announced this week, the amount of salary in each case being given after the name of the institution:

Chelsea Hospital for Women, Fulham Road, S.W., Resident Medical Officer—Salary £80 per annum, with board, &c.
Devon and Exeter Hospital, Exeter, Assistant House-Surgeon; doubly qualified, unmarried—Salary £40 per annum, with board, &c.
Great Yarmouth Hospital, House-Surgeon; doubly qualified—Salary £90 per annum, with board, &c.
Metropolitan Asylums Board, Western Fever Hospital, Seagrave Road, Fulham, S.W., Clinical Assistant for three months—Board, &c.
Wolverhampton and Staffordshire General Hospital, Wolverhampton, Resident Assistant—Board, &c.
Worcester General Infirmary, Assistant to House-Surgeon; qualified, unmarried; to act also as Dispenser—Salary £70 per annum, with board, &c.

### Non-Resident Medical Officers.

Ballymena Union (Ahoghill Dispensary), Medical Officer—Salary £105 per annum, with fees.
Borough of Cheltenham, Medical Officer of Health for the Urban Sanitary District; must reside within the district—Salary £500 per annum.
Boyle Union (Ballyfarnon Dispensary), Medical Officer—Salary £110 per annum, and fees.
Chelsea Hospital for Women, Fulham Road, S.W., Clinical Assistants.
Dental Hospital of London, Leicester Square, W.C., Dental Surgeon.
General Hospital, Birmingham, Assistant Physician. Appointment for three years—Honorarium, £100 per annum.
Grosvenor Hospital for Women and Children, Vincent Square, S.W., Chloroformist.
Liverpool Royal Infirmary, Assistant Physician.
Liverpool Royal Infirmary, Two Assistant Honorary Surgeons.
Sir Patrick Dun's Hospital, Dublin, Assistant Physician.
Sir Patrick Dun's Hospital, Dublin, Assistant Surgeon.

### PASS LISTS.

**Society of Apothecaries of London.—April, 1892.**

The following candidates passed in

Surgery.—E. G. Annis, Guy's Hospital; F. A. Arnold, London Hospital; A. H. Beardmore, Sheffield; H. J. Frederick, St. Thomas's Hospital; W. Jones, University College; M. H. Knapp, St. Mary's Hospital; P. Kotalawala, Ceylon; J. J. Mooney, Owens College, Man-

**THE STUDENT OF MEDICINE**—(*continued*).

chester; C. K. Moseley, St. Bartholomew's Hospital; S. Nesfield, Owens College, Manchester; G. A. Peake, Bristol; J. J. Powell, Cambridge University and St. Thomas's Hospital; R. W. Prentice, King's College; M. A. Saltmarsh, St. Mary's Hospital; F. J. P. Smith, St. Thomas's Hospital; T. W. Smith, King's College; W. R. Smith, King's College; F. L. Underwood, University College; and H. A. Warke, Liverpool.

Medicine, Forensic Medicine, and Midwifery.—E. G. Annis, Guy's Hospital; C. H. Broadhurst, St. Mary's Hospital; C. E. Dawes, London Hospital; A. W. Fyffe, Middlesex Hospital; J. M. James, St. Thomas's Hospital; F. K. Rider, Yorkshire College, Leeds; A. B. Rogers, Owens College, Manchester; M. A. Saltmarsh, St. Mary's Hospital; W. R. Smith, King's College; S. W. Thompson, Charing Cross Hospital; H. A. Warke, Liverpool; and S. B. Williams, St. Mary's Hospital.

Medicine and Forensic Medicine.—S. R. Lane, Middlesex Hospital.

Medicine and Midwifery.—J. B. Bate, Bristol; H. S. Chavasse, Queen's College, Birmingham; A. H. Hardcastle, Yorkshire College, Leeds; S. L. Martin, London Hospital; H. F. Ransome, Owens College, Manchester; S. A. L. Sodipo, University College; and W. E. Toyne, Edinburgh.

Medicine.—W. Ashby, Guy's Hospital; H. W. Joyce, King's College; and H. E. Mortis, Charing Cross Hospital.

Forensic Medicine and Midwifery.—C. H. A. Maggs, Charing Cross Hospital; H. E. Pittway, Middlesex Hospital.

Forensic Medicine.—J. P. Jones, London Hospital.

Midwifery.—T. E. Smurthwaite, St. Mary's Hospital.

To Messrs. Annis, Dawes, Fyffe, Frederick, J. P. Jones, Knapp, Lane, Mooney, Moseley, Nesfield, Pittway, Rider, Saltmarsh, T. W. Smith, W. R. Smith, Warke, and Williams was granted the diploma of the society entitling them to practice medicine, surgery, and midwifery.

**Royal College of Surgeons.**

The following gentlemen passed the First Professional Examination in Anatomy and Physiology for the diploma of Fellow, namely :—

Passed on Monday, May 9th.—C. A. Griffiths, M.R.C.S.Eng., of Bristol Medical School; J. M. Hall, M.R.C.S.Eng., of Belfast and London Hospital; T. W. F. Gann, M.R.C.S.Eng., of Middlesex Hospital; H. Ainsworth, of Owens College, Manchester; C. H. Bryant, of Durham University; J. S. K. Smith, M.R.C.S.Eng., of University College, Liverpool; E. L'Estrange Ledwich, of Peter Street Medical School, Dublin; C. H. Drake, of St. Bartholomew's Hospital; and G. H. Hopkins, M.R.C.S.Eng., of University Colleges Liverpool and London. Eleven candidates were referred back to their professional studies for six months.

Passed on Tuesday, May 10th.—C. H. Walker, M.R.C.S.Eng., of Cambridge University and London Hospital; C. M. Hewer, of St. Bartholomew's Hospital; A. McDougall, of Owen's College, Manchester; [illegible]t. J. Stanwell, Extramural School and Edinburgh University; E. Knight, of Owens College, Manchester; and A. W. Cuff, of St. Thomas's Hospital. Ten candidates were referred back to their professional studies for six months.

Passed on Wednesday, May 11th.—W. Roughton, M.R.C.S.Eng., of St. Bartholomew's Hospital; W. S. Melsome and W. S. Griffith, M.R.C.S.Eng., of Cambridge University and St. Thomas's Hospital; F. N. Cookson, of Middlesex Hospital; J. Griffiths, M.R.C.S.Eng., of Edinburgh and Cambridge Universities; A. H. Smith Hallidie, M.R.C.S.Eng., of London Hospital; W. G. Stone, of St. Thomas's Hospital; and L. A. Parry, of Guy's Hospital. Eight candidates were referred back to their professional studies for six months.

Passed on Thursday, May 12th.—L. J. Miskin and S. W. F. Richardson, of St. Thomas's Hospital; G. H. Cowen, of London Hospital; and C. P. White, of Cambridge University and St. Bartholomew's Hospital. Twelve candidates were referred back to their professional studies for six months.

Passed on Friday, May 13th.—F. E Greenwood and W. E. Lee, of St. Bartholomew's Hospital; J. H. Wilkes, M.R.C.S.Eng., of Cambridge University and St. Bartholomew's Hospital; E. Michels, M.R.C S.Eng., of Wurzburg, Berlin, and Kiel Universities; R. Corfe, of St. Mary's Hospital; F. C. Sprawson, J. S. Boden, and A. Bousfield, of King's College; R. J. Gladstone, of Aberdeen University and Middlesex Hospital; W. G. Mumford, L. E. V. Every-Clayton, and G. E. Manning, of Guy's Hospital; G. R. Baldwin, of St. George's Hospital; and A. Banks, M.R.C.S.Eng., of St. Thomas's Hospital. Five candidates were referred back to their professional studies for six months.

Passed on Monday, May 16th.—G. L. Eastes, of Guy's Hospital; J. H. Fisher, M.R.C.S.Eng., and C. S. Wallace, M.R.C.S.Eng., of St. Thomas's Hospital; E. B. Jones, of Charing Cross Hospital; W. J. Harris, of Cambridge University and St. Mary's Hospital; and F. Fraser, of Bartholomew's Hospital. Fourteen candidates were referred back to their professional studies for six months.

**Royal College of Physicians of Ireland.**

The following Members have been duly elected Fellows of the College: S. T. Gordon and E. E. Leemon.

At the stated examinations for the Licences of the College, held in May, 1892, the following candidates, being registered medical practitioners, were successful and have been duly admitted to their several grades in the College: For the Licence to Practice Medicine and Midwifery—W. Scatchard, M.R.C.S.Eng. For the Licence to Practice Medicine only—F. W. Spiller, L.S.A.Lond.; and A. R. Parsons, M.B. Univ.Dub. For the Licence to Practice Midwifery only—R. C. Miller, M.B.Univ.Glas., and A. L. Wykham, L.S.A.Lond.

At the stated May examination for the diploma in State Medicine, held conjointly with the Royal College of Surgeons in Ireland, the following candidates were successful: C. J. R. M'Lean, M.D.Univ.Edin.

The following has been granted the diploma of Midwife and Nurse-tender: Emily Johnina Russell Landale.

